# Technology challenges among deaf and hard of hearing elders in China during COVID-19 pandemic emergency isolation: A qualitative study

**DOI:** 10.3389/fpubh.2022.1017946

**Published:** 2023-01-05

**Authors:** Di Xu, Shiwen Ma, Chu Yan, Ziqing Zhao

**Affiliations:** School of Journalism and Information Communication, Huazhong University of Science and Technology, Wuhan, China

**Keywords:** deaf and hard of hearing elders, COVID-19, emergency isolation, technology challenges, Wuhan, qualitative study

## Abstract

Digital technology can be an effective tool to facilitate emergency assistance in a pandemic, but many deaf and hard-of-hearing elders may experience challenges in using and adopting these technologies. In the context of the second wave of the COVID-19 outbreak, this study employs a qualitative research method based on in-depth interviews to explore technology challenges among deaf and hard-of-hearing elders in China. The results showed that this group's technology challenges arose mainly from barriers to the mastery of digital technology tools, among which barriers to the use of smartphones, to the accessibility of online medical consultations, and to the presentation of health codes, were most noteworthy. For the informants, these barriers led to social isolation and technology avoidance. What's more, the expectation of individuals to adopt certain types of digital intelligence technologies can inadvertently create inequities for disadvantaged groups and exacerbate the “digital divide.” This study highlights the need for emergency management systems to be inclusive of elders with hearing loss in times of public health crises, by providing effective technology support and training to facilitate individuals' access to services and to safeguard their health, interests, and livelihood.

## Introduction

The COVID-19 outbreak caused/leads to devastating health damage in the aging population, among whom with hearing loss suffered exponential physical and psychological difficulties during the pandemic and are likely to experience extraordinary adjustment disorders as well (1–3). Despite the fact that countries are currently in a regular stage of prevention (4), the plight of older people (5), especially those with disabilities, still deserves continued attention and reflective discussion. Barriers to technology accessibility due to their own limitations and the inability of technology providers to match their demand will make this group face more challenges to survive in the digital society.

The isolating effect of the epidemic has accelerated the digital transformation of all sectors of society, with increased virtual “connectivity” and greater reliance on digital technology (6). The benefits of digital technology for the older deaf and hard of hearing communities have become a great adaptive challenge in terms of emergency assistance. There is a common and prominent psychological predisposition to technophobia among older people (7, 8), a condition that refers to anxiety and overall negative attitudes and emotional reactions to technology, its functioning or social implications (9). Many people with disabilities, especially the deaf and hard-of-hearing, are likely unable to use or normally use certain types of digital technology (due to their hearing loss), and even when using accessible digital technology are still limited by their own ability to read and write. In addition, studies from China have shown that congenitally deaf people have more difficulties interpreting written text than elders whose hearing is lost due to natural aging. The first language of this group is natural sign language, and their thinking patterns are not adapted to written expressions, and their reading and writing skills are relatively inadequate. In contrast, deaf elders with hearing loss caused by age-related degeneration also maintain the verbal thinking they acquired in earlier years and have an easier comprehension of written expressions (10). Moreover, the deaf and hard-of-hearing elders are likely to experience challenges using technology which can lead to unmet needs and limited access to services during an epidemic.

To some extent, the technology barriers and challenges experienced by deaf and hard-of-hearing elders have received attention in the context of epidemic prevention and control efforts in China. On October 20, 2020, the China Disabled Persons' Federation (CDPF), together with the Publicity Department of the CPC Central Committee, the Ministry of Civil Affairs and other departments and units, organized the “Guidelines for Social Support Services for Protection of Persons with Disabilities during Major Infectious Disease Epidemics” (11), which aims to standardize guidances, publicity instructions and social advocacy, to promote the regularization, standardization and normalization of public services related to protection of persons with disabilities under epidemics, to help persons with disabilities overcome the special difficulties and special risks brought about by epidemics, and to guarantee their equal rights to public health security. On November 24, 2020, China's State Council issued the Implementation Plan on Effectively Solving the Difficulties of Using Intelligent Technology for the Elders (12), focusing on the difficulties encountered by the elders in using intelligent technology, insisting on the parallelism between traditional service methods and intelligent service innovations, in order that the elders can better share the fruits of information development. In April 2021, China's Ministry of Industry and Information Technology issued the Notice on Improving the Implementation of Special Actions for the Aging and Accessibility Modification of Internet Applications (13), which aimed to accelerating the accessibility modification of Internet applications to help key beneficiary groups such as the elders and persons with disabilities to access and use the Internet and digital applications equally and conveniently, especially be means of issuing relevant norms and standards. China is continuously improving its social security mechanisms in order for “precise epidemic prevention and control” policies, and the government is increasingly concerned about the needs of elders with disabilities and has introduced a series of policies to promote the solutions to the issues. However, one point to be emphasized here is that the actual effectiveness of the above measures varies and depends on long-term and sustained effective outreach.

On January 23, 2020, Wuhan, the initial epicenter of the outbreak, was subjected to the most severe area-wide emergency isolation measures, which were implemented in a timely manner and achieved positive epidemic prevention results. Then in July of the following year, a new outbreak occurred in Wuhan and the government again implemented emergency isolation measures that had previously proven to be effective. Compared to the first round of outbreak prevention in 2020, the second round of outbreak prevention in 2021 utilized a large number of digital technologies to regulate the human flows by zones, in order to precisely control the spread of the outbreak. At that time, digital technology became a core survival tool, and learning to use and master it became a fundamental prerequisite for people's basic travel and access to part of essential living materials.

According to official statistics, there are 13,000 deaf and hard-of-hearing people in Wuhan (14), and more than half of the disabled population is elders (15). When both hearing impairment and aging status are combined, deaf and hard-of-hearing elders have suffered significant negative physical and psychological effects when an epidemic strikes, with some of them reporting frequent barriers to information communication and health care services (16–18). These barriers come not only from limited accessibility, but also from the technical and operational aspects that prevent access to outside help (19). This group is more vulnerable than the hearing elders, and their requests for help are often overlooked in disaster assistance. In light of this, the study explored technology challenges of deaf and hard of hearing older adults on during COVID-19 pandemic emergency isolation. This qualitative study aims to fill part of the evidence gap on the digital divide that may result from digital technologies and, as a result, technology barriers encounter by the deaf and hard-of-hearing elders since the outbreak of COVID-19, with the purpose of providing practical tactics and strategies for more vulnerable groups when they face greater risk of disparate exposure, vulnerability, and inequitable outcomes.

## 2. Methods

This study is a narrative research based on in-depth interviews through thematic content analysis that explores technology challenges among deaf and hard-of-hearing elders during emergency isolation of an epidemic and their impact. An exploratory understanding of the impact of technology challenges on deaf and hard-of-hearing elders during public health emergencies was obtained through a narrative analysis of extreme situations during special times.

### 2.1. Respondent recruitment

The subjects of this study were older deaf and hard of hearing residents aged 60 years and older from eight urban areas in Wuhan, China. The third and fourth authors of the research team are able to communicate fluently with the deaf community through local natural sign language. The research team reopened an interview list that had been used in an emergency communication study survey of disability groups between October and November 2020. At that time, the research team obtained a preliminary list of voluntary participants from the Wuhan Deaf Association, and through purposive sampling, a total of 56 deaf and hard-of-hearing elders aged 60 years and older who lived alone during the emergency quarantine period of the Wuhan epidemic were selected. The number of people who were not willing to participate in the study through initial contact was 38, the number of people who could not be reached due to no answer or change of phone number was 7, and the number of people who volunteered to participate in the follow-up study was 11, resulting in an initial list of interviewees, which was then snowball sampled to expand the list. The final number of interviewees was 13. All 13 participants on this list were revisited at the initiation of this study and individually solicited for their willingness to participate in the study again. All respondents indicated that they were willing to be interviewed a second time and capable of participating in the entirety of this impact study on technology use for people with disabilities, and all participants provided informed consent.

Among them, 5 (38.5%) were female, 7 (53.8%) were deaf, and the participants' ages ranged from 61 to 72. The participants were mostly high school graduates or less (*n* = 7) and resided centrally in urban areas (*n* = 11). 1 has “congenital” type of hearing loss and 7 have “presbycusis” type of hearing loss. Digital technology use profile: (1) 2 do not have smartphones; (2) 6 could not access outbreak information *via* smartphones; (3) 3 could not present health codes *via* smartphones; and (4) 11 could not use online medical consultations (Table 1).

**Table 1:** Basic characteristics of respondents.

**ID**	**Sex**	**Age**	**Deaf/Hard-of-Hearing**	**Education**	**Residence**	**Causes of hearing loss (congenital/** **presbycusis/** **others)**	**Have a smartphone (Y/N)**	**Ability to access outbreak information *via* smartphone (Y/N)**	**Ability to present health code *via* smartphone (Y/N)**	**Ever used an online medical consultation (Y/N)**
1	Male	63	Deaf	Undergraduate	Qingshan	Others	Y	Y	Y	N
2	Male	67	Hard-of-Hearing	Undergraduate	Jianghan	Presbycusis	Y	Y	Y	N
3	Female	76	Deaf	Elementary school	Hanyang	Presbycusis	N	N	Y	N
4	Male	69	Hard-of-Hearing	Undergraduate	Hanyang	Presbycusis	Y	N	Y	N
5	Male	66	Hard-of-Hearing	Middle school	Qiaokou	Others	Y	N	N	Y
6	Female	65	Deaf	Elementary school	Jiangan	Congenital	Y	N	Y	N
7	Female	71	Deaf	Elementary school	Jianghan	Presbycusis	N	N	N	Y
8	Male	72	Deaf	High school	Hannan	Presbycusis	Y	Y	Y	N
9	Female	70	Deaf	High school	Wuchang	Presbycusis	Y	N	Y	N
10	Female	61	Deaf	Postgraduate	Dongxihu	Presbycusis	Y	Y	Y	N
11	Male	62	Hard-of-Hearing	Junior college	Qiaokou	Others	Y	Y	N	N
12	Male	66	Hard-of-Hearing	High school	Wuchang	Others	Y	Y	Y	N
13	Male	62	Hard-of-Hearing	Postgraduate	Hanyang	Others	Y	Y	Y	N

### 2.2. In-depth interviews and data analysis

The research team followed semi-structured interview guidelines, starting the interview with an unstructured open-ended question (“Think back to a time when you used digital technology tools/platforms/software during the COVID-19 emergency isolation and tell us how you felt about it”), followed by an in-depth discussion on more specific questions one by one. With the respondents' consent, the research team used audio and video recording equipment to record all interviews, including 13 respondents' self-reported identities. Seven face-to-face interviews were conducted between May and July 2022, with each interview lasting about 2 h. Most of the interviewees used a predominantly written conversation to express their views, and four of them preferred to use natural sign language to communicate. The research team invited volunteers from the Sign Language Association, who were certified as National Professional Standard Sign Language Interpreters, to assist the research team with sign language communication, interview texts, and sign language interpretation at the interview sites. An associate professor from Yunnan Vocational College of Special Education, whose research specialty is Chinese sign language, provided guidance throughout this study's sign language transcription; a chief physician from the Department of Otolaryngology at the Maternal and Child Hospital of Hubei Province, Tongji Medical College, Huazhong University of Science and Technology, provided the medical specialty advice to the research team. The third and fourth authors in the research team, who were proficient in the sign language, were responsible for cross-checking all non-verbal information from the interviewees in the original audio and video recordings after all interviews were completed. All organized source materials were sent back to the interviewees for checking and confirmation. Following the methodological guidelines (20), this study used the thematic content analysis method to complete the data analysis and collation.

## 3. Results

Through the data analysis of in-depth interviews with 13 respondents, the impact of technology challenges on deaf and hard-of-hearing older adults in China during the emergency isolation of the epidemic was divided into two dimensions: challenges and consequences. This group's technology challenges stems from barriers to mastery of digital technology tools, specifically barriers to smartphone use, barriers to accessibility of online medical consultations, and barriers to presentation of health code. The consequences of widespread technology challenges in this group are mainly in the form of social isolation and social isolation due to the aforementioned predicament of digital technology usage, which in turn reinforces technology barriers (Table 2).

**Table 2:** Interview themes: Technology challenges and consequences.

**Challenges**	**Consequences**
Barriers to smart phone and internet use/technophobia	Difficulties using applications
	Limited access to external information
	Feeling stupid or embarrassed
	Social isolation
	Anxiety, depression, and other psychological problems
Barriers to the accessibility of online medical consultations	Inability to obtain appointment and/or meet with medical care team
Barriers to using Health Code program	Inability to enter/exit public places
	Unmet basic necessities of life

### 3.1. Barriers to smartphone use and internet use

In tackling the epidemic, China has accelerated its digital development process, with intelligent services and Internet technologies flourishing. However, the elders has been slower to adapt to the changes. According to the China Internet Development Statistics Report, Chinese Internet users aged 60 and above account for 12.2% of the overall population in 2021. More than half of the respondents said they could not use the Internet skillfully and had difficulties with smartphone applications, and about 15.3% said they did not have a smartphone (21). During the emergency isolation period of the epidemic, because they do not use smart phones or APPs, the deaf and hard-of-hearing elders have relatively limited access to external information. Most deaf and hard-of-hearing elders may not be able to keep abreast of and understand the development situation of the epidemic, and their knowledge of basic epidemic prevention is generally inferior, thus easily forming the psychological characteristics of herd panic or stubbornness and conceit, which is not conducive to the implementation of epidemic prevention measures.

In addition, family and friends are the main components of social relationships for the older deaf and hard-of-hearing group, and their ability to communicate with family or friends for emotional support, crisis assistance, and opportunities for social participation has a significant impact on the lives of this group. With limited opportunities for communication and expression, many elders with hearing loss may be lacking in emotional support and comfort. They are unable to talk about their worries and anxieties about the epidemic itself, and are more likely to develop negative emotions such as nervousness, fear, and loneliness. While digital technology might be a virtual bridge for hearing people to maintain interpersonal communication in times of social isolation, for deaf and hard-of-hearing elders, digital technology may not only be the cause of emotional impairment but also does not provide the functionality it should. In this way, it appears that digital technology becomes an enabler of reverse social isolation in the case of the deaf and hard-of-hearing elders.

“*For information about the epidemic, I only watch sign language broadcasts on the TV news, I don't go online or look at the information on the social media apps on my phone” (Interviewee 4)*.

“*You all use things like WeChat (instant messaging software) to check epidemic information and emergency notifications and so on, but I don't very well use my phone. I'm talking about a smartphone, the operation, you know, it's too complicated. Obviously I don't go online either” (Interviewee 9)*.

“*Community workers are contacting everyone in the WeChat group, and I'm really not good at using WeChat, and I can't see too many outbreak notifications in the WeChat group” (Interviewee 6)*.

“*I couldn't go out during the quarantine period, so I couldn't see my friends or find anyone to talk to, and I had no entertainment to get rid of my loneliness. People said I could chat online, but how could I do that? I typed very slowly and my phone screen was very small, so I felt more and more depressed when I used it” (Interviewee 2)*.

“*I was placed in quarantine once. I was so worried when I was under quarantine observation. I prayed every day that the infection would never be confirmed. My children and I had video calls at first, once a day. Watching them through the electronic screen, I gestured and gestured while I was sad, and then I deliberately told them to stop talking every day and that I would call them back if something happened” (Interviewee 13)*.

### 3.2. Barriers to the accessibility of online medical consultations

The epidemic prevention and control has promoted the development of “Internet+” services in China, such as online medical consultation and online procurement of daily necessities. For most deaf and hard-of-hearing elders, online medical consultation or online appointment procurement is problematic, and it is hardly possible for them to effectively obtain the basic necessities of life. Due to the requirements of epidemic prevention and control restricting daily visits from relatives and friends (Interviewee 8), the deaf and hard-of-hearing elders are unable to get help through acquaintances and rely only on community service staff for assistance in purchasing medicine and medical care, in which not only do they face obstacles in communication, but also may increase the psychological burden of the deaf and hard-of-hearing elders and induce anxiety, depression and other psychological problems.

“*I have high blood pressure and I've been taking antihypertensive pills, and I ran out of antihypertensive pills when I was isolated and didn't know how to buy them. They all say buy medication online, buy medication online, I don't even have a smartphone, how can I buy them, the pills? I want to get permission from the neighborhood council to buy medicine out of the community, and I don't know what online process to go through, and which software to buy medicine on” (Interviewee 7)*.

“*During the epidemic I could not buy medicine, hospitals and pharmacies were closed, and access to the community was prohibited, so I could not buy if I was worried about the problem. I was dying of anxiety, and eventually, a community sign language volunteer helped me by teaching me to consult and prescribe medication online” (Interviewee 5)*.

### 3.3. Barriers to using Health Code program

Health Code is one of the highlights of China's digital anti-epidemic efforts, bringing great convenience to the management of human flows and the prevention of epidemic transmission. The Health Code is a small program that can be installed on smartphone clients (e.g., WeChat or Alipay), which enables individuals to authenticate and fill in their health status and then use it as an electronic credential for local access. By comparing the data with cell phone roaming trajectory and close contacts, the platform can verify the information filled in by individuals independently and manage the information of administrators in an accurate and dynamic method. With the health code as the identification system, the healthy personnel could pass with the health code in the applicable area (22).

According to official data, 27.2% of Chinese non-Internet users consider that not having access to the Internet will bring various kinds of inconvenience to their lives, and the inability to enter and exit some public places without a “health code” ranks first. For many in the older deaf and hard-of-hearing communities, the lack of proficiency in mastering online skills brought about by aging and a series of derived barriers from hearing impairment make their digital survival a more serious challenge. Worthy of mention is the fact that deaf and hard-of-hearing elders may not be able to self-identify their health using the health code embedded in the smartphone Apps due to a number of factors such as not having a smartphone or not being proficient in using various smartphone functions, which makes their mobility somewhat limited. This important initiative to digitize China's fight against the epidemic has unexpectedly created some difficulties in the use of vulnerable groups.

“*To be honest I don't know how to check the health code on my phone, I tried to learn and tried many times but I can't learn it, it makes me look stupid. I don't want to click on this App again. But you need a health code to go anywhere, and of course you can't go anywhere without a green code” (Interviewee 11)*.

“*I didn't have the opportunity to get a good education as a child. It was thanks to my daughter's foresight that I was taught to set up my health code in advance when I was in isolation. I don't know what I would have done without my daughter's help” (Interviewee 6)*.

“*Now you have to show a health code to go anywhere out of the community, and I don't have a smartphone and I don't know how to operate it. Thanks to the community, the staff helped me to issue a paper health certificate so I could go out” (Interviewee 3)*.

## 4. Discussion

Based on the China context, this study is framed at the intersection of disability studies and digital technology studies, exploring both the impact of technology challenges and barriers on deaf and hard-of-hearing elders from a technological perspective and insisting on observing the personal feelings and experiences of deaf and hard-of-hearing elders during the emergency isolation of the COVID-19 epidemic. To this end, this study highlights the subjective experience and subjectivity of people with disabilities and focuses on the digital technology use practices of deaf and hard-of-hearing elders during the epidemic through a qualitative research method of in-depth interviews. In particular, the smartphone is a tool for digital technology use, the health code is a carrier of digital technology form, and the online medical consultation is a technical expression of digital to good, one of the bright spots of digital anti-epidemic (23). Based on this, this study examines the effects of technology challenges during COVID-19 emergency isolation on the deaf and hard-of-hearing elders.

China has about 27.8 million people with hearing impairment, accounting for one-third of the total number of people with disabilities in the country, and is the country with the largest number of people with hearing disabilities in the world (14). China also has a very large base of older people, and an official statistic shows that as of December 2020, 11.2% of China's population was aged 60 and above, still the main group of non-Internet users. Due to their physiological hearing deficits, people with hearing loss are unable to use or function normally with certain types of digital technology, and most deaf and hard-of-hearing groups are native speakers of sign language and use written or spoken language as a second language, and are limited in their ability to read and write when using digital technology. Many elders have difficulties in accessing and using digital technology due to factors such as physical and mental decline. Beyond the experience of using technology, digital technology is a relatively new thing for hearing elders to master in a basic way through repeated use over time. Beyond the experience of using the technology, digital technology is still a new thing for most hearing elders to master in a basic level through repeated use over time. Proficiency in digital technology is an almost impossible task for the vast majority of deaf and hard-of-hearing elders, especially for those with congenital hearing disabilities and low levels of education (Interviewee 6, Interviewee 3). When such digital technologies that could not be mastered through self-learning, or even certain types of digital technologies that are mandatory for emergency purposes, become artificial “digital rift,” it could be concluded that the existence of such technologies inadvertently raises the difficulty for the disadvantaged groups to benefit from equitable rights and interests, and intensifies the formation of “digital divide.”

During the COVID-19 epidemic, the isolation of living spaces limited face-to-face communication opportunities between the deaf and hard-of-hearing elders and their family and friends, and the deaf and hard-of-hearing elders were unable to use online communication devices and software proficiently due to their physical disadvantages and fear and anxiety about using technology (Interviewee 6), and communicated with their family and friends online less frequently or even disconnected (Interviewee 7, Interviewee 13). Findings suggest that the social ties of the deaf and hard-of-hearing elders during the special period, such as kinship, regional and occupational ties, were no longer tight, and social support networks were forced to be disrupted or even completely broken, which made it difficult for them to receive adequate emotional sustenance during the epidemic. In particular, older adults who are congenitally deaf grew up in an environment where the compulsory education system was not well established more than half a century ago, and their adolescence was characterized by the unavailability of a quality K12 education. The objective factors mentioned above lead to the weak reading and writing ability of these groups, and their later interpersonal communication is mostly confined to a small-scaled community, with limited social contact accomplished through natural sign language. In this case, the technological feedbacks support from their children somewhat alleviate the digital survival predicament (Interviewee 6), but due to the limitations of their education level, they will still be relegated to a branch of the population that is the slowest to complete the acquisition of digital skills during the particular period of interpersonal communication breakdowns. The home isolation during the epidemic caused the elders with hearing loss to move from a group-dependent living space to a highly individualized living space. The living space was compressed and the psychological space was divided, leaving the elders with hearing loss in a double isolation of living space and psychological space during the epidemic, and this isolation was reinforced by technology challenges. From another perspective, the social isolation during the epidemic was also an important cause of technology barriers among the deaf and hard-of-hearing elders. Social isolation cut off this group from learning to use digital technology, which made them more vulnerable to unadaptation to new digital technologies and unmet needs during the epidemic due to technology challenges and barriers. In this way, it appears that digital technology becomes an enabler of reverse social isolation in the case of the deaf and hard-of-hearing elders.

## 5. Limitations

There are potential limitations of this study to note. First, the sample size used in this study is small and the results obtained could not represent the full sense of the situation. Second, this study is conducted through retrospective interviews and the respondents may have some memory bias, which could have some impact on the results. Finally, the study did not include individuals over age 76, who are likely to experience greater barriers in technology adoption.

## 6. Conclusion

This study focuses on the impact of technology challenges on the deaf and hard-of-hearing elders during epidemic isolation, how they respond to intelligent technology use, and their perceptions of new digital technologies, and reveals the multifaceted phenomena and diverse experiences of coping technology barriers in this population. Digital technology can be an effective tool to facilitate the provision of emergency assistance in the event of a pandemic, but the deaf and hard-of-hearing elders is likely to experience technology challenges which can lead to serious consequences including limited access to healthcare and essential services as well as social isolation. This study not only attempts to reveal the real existence of this phenomenon, but also expects to shed light on social issues of concern through the presentation of the effects of technology barriers in this particular group. Moreover, this study emphasizes the necessity for emergency management systems to consider the wellbeing of the deaf and hard-of-hearing elders and to provide effective technical support and training to promote social support services for this group and to safeguard the health, interests, and livelihoods of individuals.

## Data availability statement

The original contributions presented in the study are included in the article/supplementary material, further inquiries can be directed to the corresponding author.

## Ethics statement

Ethical review and approval was not required for the study on human participants in accordance with the local legislation and institutional requirements. The patients/participants provided their written informed consent to participate in this study.

## Author contributions

Conceptualization, methodology, writing—review and editing, and supervision: DX. Investigation, data curation, and writing—original draft preparation: SM, CY, and ZZ. Project administration and funding acquisition: DX and CY. All authors have read and agreed to the published version of the manuscript.
